# Deep-sea mystery solved: astonishing larval transformations and extreme sexual dimorphism unite three fish families

**DOI:** 10.1098/rsbl.2008.0722

**Published:** 2009-01-20

**Authors:** G.David Johnson, John R. Paxton, Tracey T. Sutton, Takashi P. Satoh, Tetsuya Sado, Mutsumi Nishida, Masaki Miya

**Affiliations:** 1 Division of Fishes, National Museum of Natural History, Smithsonian InstitutionWashington, DC 20560, USA; 2 Ichthyology, Australian MuseumSydney, New South Wales 2010, Australia; 3 Virginia Institute of Marine ScienceGloucester Point, VA 23062, USA; 4 Marine Bioscience, Ocean Research Institute, University of TokyoNakano-ku, Tokyo 164-8639, Japan; 5 Zoology, Natural History Museum and InstituteChuo-ku, Chiba 266-8682, Japan

**Keywords:** Cetomimidae, Megalomycteridae, Mirapinnidae, ontogenetic transformation, sexual dimorphism, whalefishes

## Abstract

The oceanic bathypelagic realm (1000–4000 m) is a nutrient-poor habitat. Most fishes living there have pelagic larvae using the rich waters of the upper 200 m. Morphological and behavioural specializations necessary to occupy such contrasting environments have resulted in remarkable developmental changes and life-history strategies. We resolve a long-standing biological and taxonomic conundrum by documenting the most extreme example of ontogenetic metamorphoses and sexual dimorphism in vertebrates. Based on morphology and mitogenomic sequence data, we show that fishes currently assigned to three families with greatly differing morphologies, Mirapinnidae (tapetails), Megalomycteridae (bignose fishes) and Cetomimidae (whalefishes), are larvae, males and females, respectively, of a single family Cetomimidae. Morphological transformations involve dramatic changes in the skeleton, most spectacularly in the head, and are correlated with distinctly different feeding mechanisms. Larvae have small, upturned mouths and gorge on copepods. Females have huge gapes with long, horizontal jaws and specialized gill arches allowing them to capture larger prey. Males cease feeding, lose their stomach and oesophagus, and apparently convert the energy from the bolus of copepods found in all transforming males to a massive liver that supports them throughout adult life.

## 1. Introduction

**Figure 1:**
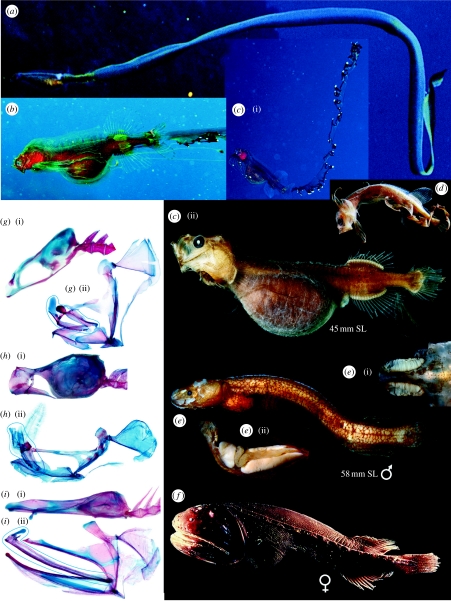
Life stages and selected skeletal elements of cetomimid whalefishes. (*a*) *Eutaeniophorus festivus* postlarva, BSKU 51970, 56 mm SL, approximately 816 mm TL, photo courtesy of Masanori Nakamachi, ‘Sea Fishes of Japan’ © YAMA-KEI Publishers Co., Ltd. *P. brevis*?: (*b*) postlarva, Cozumel, Mexico, photo courtesy of Donald Hughes; (*c*) postlarva, KPM NI13654: (i) photo courtesy of Yasuhiro Morita, (ii) photo courtesy of Sandra Raredon, USNM; (*d)* larva, MCZ 59910, 13 mm SL, photo courtesy of Chris Kenaley, © President and Fellows of Harvard College; (*e*) *Ataxolepis apus* adult male, USNM 391648: (i) dorsal view of nasal organs, (ii) lateral view of viscera, enlarged liver on left, enlarged testes dorsal and ventral right, intestine middle right. (*f*) *Gyrinomimus* sp., juvenile female, NE Pacific, photo courtesy of Bruce Robison, MBARI. (*g*(i), *h*(i), *i*(i)) Cranium and anterior vertebrae, and (*g*(ii), *h*(ii), *i*(ii)) left jaws, palatine arch, suspensorium and operclular bones of (*g*) *E. festivus* postlarva, USNM 391655, 60 mm SL, (*h*) *A. apus* adult male, USNM 391649, 58 mm SL and (*i*) *C. regani* female, USNM 391657, 93 mm SL, respectively. Blue ‘ovals’ enclose maxillae, premaxillae and rostral cartilage, which, in (*h*(ii)) are fused to each other and to broken nasals. (*g*–*i*) Photo courtesy of G.D.J.

New specimens from collecting expeditions continue to provide insights into the many mysteries of the Earth's largest ecological habitat, the midwaters of the deep sea between the sunlit surface waters and the bottom. The Cetomimidae (whalefishes), one of the most speciose bathypelagic fish families (nine genera, 20 species), were described by Goode & Bean (1895). There are no larvae among the 600+ whalefish specimens (26–408 mm standard length (SL)), collected below 1000 m; all sexually mature individuals are females (Paxton 1989). Adults have whale-shaped bodies, tiny eyes, huge horizontal mouths, cavernous lateral-line canals, and lack pelvic fins and external scales (figure 1*f*). The Mirapinnidae (hairyfish and tapetails) were described as a new order by Bertelsen & Marshall (1956) and comprise five species in three genera; they lack scales and lateral lines, have large mouths with almost vertically oriented jaws and pelvic fins (figure 1*a*–*d*). The hairyfish, known from a single specimen, is uniquely characterized by a dense covering of hair-like outgrowths over the head, body and fins. Tapetails have the skin of the caudal fin prolonged into a long ribbon-like streamer that may extend nine times the body length. All 120 mirapinnid specimens (5–56 mm) are sexually immature, and all but four were collected in the upper 200 m. The Megalomycteridae (bignose fishes), described by Myers & Freihofer (1966), comprise four monotypic genera. These small (34–68 mm), elongate fishes have huge nasal organs, small, horizontal mouths with immobile upper jaws, non-overlapping, mosaic scales and lack pelvic fins (figure 1*e*). Most of the 65 specimens were collected below 1000 m and all those examined are males (Paxton 1999).

Gosline (1971) first recognized that these three families (currently placed in the order Stephanoberyciformes) are closely related and suggested that megalomycterids could be macrosomatic male cetomimids. Robins (1974) mentioned that some ‘mirapinniforms’ are pre-juvenile cetomimids, without any supporting evidence. Miya *et al*. (2003) found the mitochondrial genome of a mirapinnid specimen to be almost identical with that of a whalefish, differing in only seven among 16 500 base pairs sequenced. The striking morphological differences between these two families and absence of a voucher specimen for the mirapinnid caused two of us to question these results (Paxton & Johnson 2005), even though some meristic data show striking concordance among species pairs from each family.

**Figure 2:**
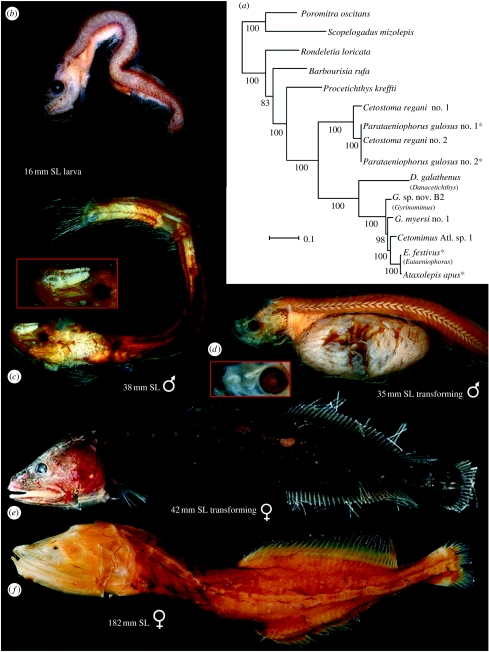
(*a*) ML tree derived from analyses of whole mitogenome sequences from 15 specimens using RAxML v. 7.0.4. Numerals beside internal branches indicate bootstrap values (only 50% and above are shown) based on 1000 replicates. Scale indicates expected number of substitutions per site; red asterisks, larvae; blue asterisk, male. Long-finned whalefish *C. regani* Zugmayer, 1914: (*b*) USNM 391563; (*c*) MCZ 60609 (inset, enlarged nasal organ); (*d*) BMNH 1957.7.20.1.00, holotype of *P. gulosus* (inset, elongate nasal rachis); (*e*) USNM 392646; (*f*) USNM 391656. Photo courtesy of S. Raredon and G.D.J.

Excellent new Gulf of Mexico megalomycterid specimens with closing-net data that placed them together with the cetomimids at 1500–2000 m depth led us to re-examine the problem. We discovered that the holotype and only known specimen of the megalomycterid *Megalomycter teevani* is actually a transforming mirapinnid, as evidenced by the remains of three small pelvic-fin rays, a slightly oblique mouth, a gut full of copepods and still-developing nasal organ anlagen. Subsequently, we found that the holotype of *Parataeniophorus gulosus* (figure 2*d*), one of the few mirapinnids collected at depths greater than 200 m, is in a similar, but earlier, state of transition. The identity of mirapinnids as larval megalomycterids was thus established. Fortuitously, a transforming specimen of the cetomimid long-finned whalefish *Cetostoma regani* (figure 2*e*) was captured shortly thereafter.

## 2. Material and methods

Clearing and staining procedure follows Dingerkus & Uhler (1977). Collection acronyms follow Eschmeyer (1998). SL=standard length; TL=total length.

DNA from 34 individuals of all five whalefish ‘families’ representing 10 genera and 16 presumed species plus two melamphaids as outgroups was analysed (see table S1 in the electronic supplementary material, including GenBank numbers). Whole mitochondrial genome (mitogenome) sequences for nine species were newly determined and used with an additional six such sequences available from GenBank (total 15 species). The mitogenomes (approx. 16 500 bp) were determined using a combination of long and short polymerase chain reactions and direct cycle sequencing techniques following the methods of Miya & Nishida (1999). For the remaining 21 individuals, we determined partial sequences of the 16S ribosomal RNA (rRNA) gene (approx. 575 bp).

Unambiguously aligned mitogenome sequences from 15 specimens were divided into five partitions (first, second and third codon positions of the 13 protein-coding, rRNA and tRNA genes; total=15 886 positions) and subjected to the partitioned maximum-likelihood (ML) analysis using RAxML v. 7.0.4 (Stamatakis 2006). We estimated the best-scoring ML tree using a general time reversible (GTR)+gamma model of sequence evolution with 1000 bootstrap replicates. The resulting ML tree was then used as a backbone constraint (−*r* option in RAxML) for subsequent ML analysis using unambiguously aligned, partial sequences of the 16S rRNA gene from all 36 specimens. We similarly estimated the best-scoring ML tree using a GTR+gamma model of sequence evolution with 1000 bootstrap replicates. More details of the DNA methods are in the electronic supplementary material.

## 3. Results and discussion

We identified three specimens in transition from larval/juvenile stage to adult. The 41.7 mm *C. regani* taken in an open net fished to a depth of 5110 m in the southeastern Atlantic is a late transforming female that retains only 3–4 of the 8–10 pelvic-fin rays found in the larvae; pelvic-fin rays are lacking in the other 184 female specimens of this most common whalefish (figure 2*e*). This species has uniquely high dorsal- and anal-fin ray counts of 26–37 compared with 11–22 rays for all other taxa in the family, allowing links with *P. gulosus* larvae/postlarvae and *Cetomimoides parri* males (figure 2*c*). The 35 mm holotype of *P. gulosus* (figure 2*d*) collected in a closing net between 700–1400 m is an early transforming specimen with a full complement of 10 pelvic-fin rays, moderately long jaws and a gut full of copepods. Although the nasal organ is incompletely developed, the elongate, thickened median rachis (figure 2*d* inset) indicates that the individual would have developed into a male. The 34 mm holotype of *M. teevani* described above was caught in an open net fished to a depth of 1650 m. Histology of the gonad reveals good spermatogenic tissue with pre-spermatids (H. G. Moser 2006, personal communication).

A detailed osteological description of the three life stages is beyond the scope of this paper, but images of the various stages shown in figures 1 and 2 illustrate the amazing ontogenetic transformations that result in extraordinary sexual dimorphism. These transformations include major changes in jaw length, depth and angle, and concomitant radical modifications of the suspensorium and angle of attachment of the skull to the vertebral column (figure 1*g*–*i*). Females develop taxon-specific gill arch structure and males exhibit hyperossification of most bones. Of the latter, most remarkable are fusion of the first vertebra to the occiput and of the hypertrophied nasal, lacrimal and upper jawbones (figure 1*h*), our first clue that males do not feed.

Transformed males lack an oesophagus and stomach, but retain a vestigial, thin-walled intestine containing copepod tests; a massive liver and paired gonads fill the peritoneal cavity (figure 1*e*(ii)). Most of the largest juveniles have a gut swollen with copepods (40–200+, *n*=6) visible externally in life as a swollen orange bulge. This bolus of copepods must provide the nutrition required to generate the large liver that sustains the male through the rest of its life. This is unnecessary in females that continue to feed and may reach more than 40 cm. The transforming female *Cetostoma* has neither a gut full of copepods nor a massive liver.

The most striking feature of the larvae is the streamer that grows from the caudal-fin rays, just visible in the smallest 4–5 mm larvae, but extending an estimated 75 cm in the largest postlarva photographed (figure 1*a*). The two largest photographed specimens (figure 1*a*,*c*), both with copepod-gorged guts, lost their streamers during capture. The most striking streamer is that of *Parataeniophorus brevis*, with ornamentation reminiscent of a siphonophore (figure 1*b*,*c*). One can only speculate regarding the possible advantages and disadvantages of this remarkable appendage in feeding versus predator avoidance. Videos of live female whalefish show that their locomotion involves both rapid swimming with sinusoidal body waves and slow swimming with undulations of dorsal and anal fins (see video A in the electronic supplementary material).

In recent years, additional tissues have become available, with the total mitogenomic analyses that provided the ML tree (figure 2*a*) from one male specimen, three larvae representing two species and six species of females in five genera. The linking of larval *P. gulosus* with *C. regani* is confirmed, with an ML tree based on 16S rRNA analyses (see figure S1 in the ESM) including two larvae and nine females of this species. Larval *Eutaeniophorus* and male *Ataxolepis* are embedded within the genera *Cetomimus* and *Gyrinomimus.* With outgroups of the stephanoberyciform Rondeletiidae and Barbourisiidae, the generic relationships of the cetomimids largely follow those proposed by Paxton (1989). The basal position of *Procetichthys* is confirmed, while notable differences include the more basal position of *Cetostoma* and the paraphyly of *Gyrinomimus*. Further analyses combining morphologic and genetic data are planned, while tissues from additional genera and larvae are needed. With the synonymy of the three families confirmed, the next challenge is to link the three life stages of each species. Meristic data establish *Mirapinna esau* as the postlarva of *Procetichthys kreffti* and suggest that *Parataeniophorus bertelseni* is the larva of *Ditropichthys storeri.*

Although remarkable ontogenetic transformations occur in a few other deep-sea fish families (e.g. Giganturidae), and prominent sexual dimorphism is widespread among vertebrates, the extraordinary combination of both that we have documented here for the whalefishes is unparalleled within Vertebrata.

## Supplementary Material

Methods, results, discussion and acknowledgements Includes legends for Supplementary Figure S1 and Supplementary Video A.
