# Supplementary material for: Deep-sea mystery solved: astonishing larval transformations and extreme sexual dimorphism unite three fish families
Source: Biol Lett. 2009 Jan 20;5(2):235–9. doi: 10.1098/rsbl.2008.0722 (PMC2667197; doi:10.1098/rsbl.2008.0722)
Supplement: Methods, results, discussion and acknowledgements — Includes legends for Supplementary Figure S1 and Supplementary Video A. [file rsbl20080722s14.doc]

SUPPLEMENTARY METHODS, RESULTS, DISCUSSION AND ACKNOWLEDGEMENTS

*Additional Methods*

Video Analysis

Calculations of swimming times were made on uncompressed video files.

Specimens and DNA Extraction

We used 34 individuals of all five whale fish “families” representing 10 genera and 16+ species plus two melamphaids as outgroups in this study (Table S1). A portion of the epaxial musculature (*ca*. 0.25 g) was excised from fresh or frozen specimens of each individual and preserved immediately in 99.5% ethanol. Total genomic DNA was extracted using a Qiagen DNeasy tissue kit (Qiagen, Hilden, Germany) in accordance with the manufacturer’s protocol.

PCR and Sequencings

We newly determined whole mitochondrial genome (mitogenome) sequences for nine species and used them with an additional six such sequences available from the databases (total 15 specimens). The mitogenomes were amplified and sequenced using a combination of long and short polymerase chain reactions (PCRs) and direct cycle sequencing techniques (Miya and Nishida, 1999). The reactions were carried out as previously described (Miya and Nishida, 1999). We used six fish-versatile long-PCR primers in the following four combinations to amplify the entire mitogenome (L2508-16S + H12293-Leu; L2508-16S + H15149-CYB; L8343-Lys + H1065-12S; L12321-Leu + S-LA-16S-H; for location and sequences of these primers, see Miya and Nishida, 1999, 2000; Inoue et al., 2000, 2001; Kawaguchi et al., 2001; Ishiguro et al., 2003). The long-PCR products were diluted in TE

buffer (1:19) for templates of subsequent short PCRs. A total of >150 fish-versatile PCR primers were used in various combinations to amplify the contiguous, overlapping segments of the entire mitogenome. A list of the short PCR primers used for each species is available from M.M. upon request.

Double-stranded PCR products, purified using an ExoSAP-IT (USB Corp., Cleveland, OH), were subsequently used for direct-cycle sequencing with dye-labeled terminators (Applied Biosystems, Foster City, CA). The primers used were the same as those for PCR. All sequencing reactions were performed according to the manufacturer’s instructions. Labeled fragments were analyzed using a Model 3130*xl* DNA sequencer (Applied Biosystems).

For the remaining 21 individuals, we determined partial sequences of the 16S ribosomal RNA (rRNA) gene using a primer pair of L2510-16S + H3084-16S (for sequences of these primers, see Miya and Nishida, 1999). Protocols for PCR and direct sequencings were the same as those for whole mitogenome sequences (see above).

Phylogenetic Analysis

The DNA sequences were edited, assembled, and analysed with EditView ver. 1.0.1, AutoAssembler ver. 2.1 (Applied Biosystems), and DNASIS ver. 3.2 (Hitachi Software Engineering, Tokyo, Japan). Thirteen protein-coding, two rRNA, and 22 tRNA gene sequences for 15 specimens were manually aligned using MacClade ver. 4.08 (Maddison and

Supplementary Table S1. List of specimens used in this study

—————————————————————————————————

species accession number voucher specimen

—————————————————————————————————

Melamphaidae (outgroup)

*Poromitra oscitans* AP002935 CBM-ZF 10631

*Scopelogadus mizolepis* AP002934 ——

Rondeletiidae

*Rondeletia loricata* AP002937 ——

Barbourisiidae

*Barbourisia rufa* AP010879 NMV A26287002

Cetomimidae

*Cetomimus* Atl. sp.1 AP010881 AMS I34481001

*Cetomimus craneae*?*AB462437 MCZ 165975

*Cetomimus* sp. #1*AB462445 MCZ 164247

*Cetomimus* sp. #2*AB462449 MCZ 163189

*Cetomimus* sp. #3*AB462448 MCZ 163191

*Cetomimus* sp. #4* AB462452 MCZ 158584

*Cetomimus* sp. #5* AB462455 ASIZ P0070023

*Cetomimus* sp. #6*AB462442 MCZ 164796

*Cetomimus* sp. #7*AB462441 MCZ 164246

*Cetomimus* sp. #8*AB462443 MCZ 164792

*Cetomimus* sp. #9*AB462439 MCZ 164784

*Cetostoma regani* #1 AP010882 MCZ 164250

*Cetostoma regani* #2 APO04423 ——

*Cetostoma regani* #3*AB162447 MCZ 163207

*Cetostoma regani* #4*AB462453 MCZ 158722

*Cetostoma regani* #5*AB462446 MCZ 163208

*Cetostoma regani* #6*AB462444 MCZ 164251

*Cetostoma regani* #7*AB462450 MCZ 161539

*Cetostoma regani* #8*$ AB462440 MCZ 164791

*Cetostoma regani* #9*AB462451 MCZ 161538

*Danacetichthys galathenus* AP002936 ——

*Gyrinomimus bruuni* sp. group*AB462438 MCZ 161493

*Gyrinomimus* sp. nov. B2 AP010883 UW 113191

*Gyrinomimus* sp.nov. L*AB462436 MCZ 164174

*Gyrinomimus myersi* #1 AP010884 ZMUB 19723

*Gyrinomimus myersi* #2*** AB462454 ZMUB 19722

*Procetichthys kreffti* AP010880 ZMUB 19724

Mirapinnidae

*Eutaeniophorus festivus* AP010885 USNM 391548

*Parataeniophorus gulosus* #1 AP010886 USNM 391563

*Parataeniophorus gulosus* #2 AP004424 ——

*Parataeniophorus brevis**AB462456 USNM 391560

Megalomycteridae

*Ataxolepis apus* AP010887 MCZ 164705

—————————————————————————————————

*Specimens with only 16S rDNA sequences determined.

$ Presumably this specimen, as there was a labeling error at sea.

Maddison, 2000). All sequences from L-strand-encoded genes (ND6 and eight tRNA genes) were converted to complementary strand sequences. Deduced amino acid sequences were used for alignment of the protein-encoding genes. All positions including gaps and stop codons were excluded. Totals of 11361, 2540, and 1985 nucleotide positions were unambiguously aligned (total = 15886 positions).

Partitioned maximum likelihood (ML) analysis was conducted with RAxML ver. 7.0.4 (Stamatakis 2006) for the unambiguously aligned 15 whole mitogenome sequences. We set five partitions, assuming that functional constraints on sequence evolution are more similar within codon positions (or types of molecule) across genes than across codon positions (or types of molecule) within genes. The GTR model, with variable sites assumed to follow a discrete gamma distribution (GTR + G; Yang 1994), was used for all partitions according to the recommendations in a manual of RAxML ver. 7.0.4 (Stamatakis, 2006). We reconstructed an ML tree, simultaneously conducting bootstrap analysis for the best-scoring tree topology with 1000 replicates.

The best-scoring tree topology was then used as a backbone constraint (-r option in RAxML) for subsequent partitioned ML analysis using partial sequences of the 16S rRNA gene. We extracted relevant portions of the sequences from the above alignment based on whole mitogenomes and realigned the extracted sequences with an additional 21 partial sequences (total = 36 sequences). Unambiguously aligned sequences (575 nucleotide positions) were subjected to partitioned ML analysis as described above.

*Additional Results and Discussion*

Whalefish Videos

Videos of two female long-finned whalefish, *Cetostoma regani*, swimming at depths of 1450 and 1640 on 19 November 2004 and 5 April 2006 respectively, were taken in Monterey Canyon off the coast of California by research scientists from the Monterey Bay Aquarium Research Institute using the ROV *Tiburon* (video A). These first images of living whalefish provide new insight about both the swimming modes and deep-sea life of this species.

Bathypelagic fishes in general are considered lethargic, slowly swimming forms that wait for food to swim or drift by (Marshall 1971). A concise summary of the significant literature on different swimming modes is provided in the textbook of Helfman *et al*. (1997). Westneat *et al.* (1998) detailed the fast-start escape response in primitive fishes. Moore (2002) described video images of three gigantactinid anglerfish of less than 15 seconds each at 5000 m. Luck & Pietsch (2008) described in detail the swimming of an oneirodid anglerfish based on 24 minutes of video images at 1474 m.

Video Segment 1 (2004) includes 2.5 seconds of fish images, while Segment 2 (2006) includes 33 seconds of fish images; Segment 3 is a slow motion of the latter portion of Segment 2. Images of both individuals clearly show the long-finned whalefish swimming, with two distinct swimming modes. Rapid undulatory swimming due to sinusoidal waves passing down the body is seen in Segment 1 and the last portion of slow motion Segment 3. In Segment 1 the fish glides into view, has two periods of rapid swimming separated by a very brief glide, and then glides out of view. The undulation appears most evident in the posterior half of the body. The fish appears close to the ROV, as the bright lights bring out the orange colour and the rapid swimming suggests it is aware of the ROV presence. In Segment 2 the fish appears some distance from the ROV and initially is brought closer by the camera lens; the colour is orange-brown and the light does not appear as bright. Rapid swimming only occurs near the end of the segment, when the fish presumably becomes aware of the ROV and swims away in a burst of speed.

The fast start near the end of slow motion Segment 3 is the classic C-start described by Westneat *et al.* (1998), with both head and tail bending to the right. The camera angle does not allow estimation of the total extent of the maximum curvature. The fish swims out of sight after only two undulations and then reappears some body lengths away 1.7 seconds after burst swimming commenced, at the end of Segment 2. Although the fish appears to be swimming in the uncompressed file, details are impossible to discern.

While no dorsal view of swimming is available for confirmation, this long-finned whalefish appears to utilise the subcarangiform type of rapid swimming, with undulations more pronounced posteriorly combined with a low aspect ratio tail. This type of swimming, with a low, flexible tail, is better suited for rapid acceleration from a dead start (Helfman *et al.* 1997), as clearly seen here. Two pale scars on the lateral side of the body in Segment 2 suggest that there are predators in the bathypelagic zone from which rapid acceleration is an advantage. The stinging cells of siphonophores or medusae, both common at these depths, could be responsible for such scars (B. Robison, pers. com. Oct., 2008).

A second type of slow swimming that involves undulation of the dorsal and anal fins is clearly visible in Segments 2 and 3. The first few rays of each fin do not move as much as the remaining rays. In the long-finned whalefish, the first three to four rays of each fin are unbranched, shorter, and heavier than the remaining branched rays (Paxton 1989). Although both fins undulate together, undulations of the dorsal fin are more pronounced than those of the anal fin. In this individual, the rays of the dorsal fin appear longer than those of the anal fin. Perhaps the curtain-like folds of membrane between the last 10 rays of the anal fin, unique to this species of whalefish (Paxton 1989), inhibit the undulations of this fin. The dorsal and anal fins are visible clearly for 24.5 seconds of Segment 2; fin undulation takes place for 7.0 seconds, or 28.6% of the time. This species has no trace of a swimbladder (Marshall 1960), and perhaps minimal swimming with fin undulations is of aid in retaining the same depth in the water column.

Luck & Pietsch (2008) found the anglerfish *Oneirodes* sp. at 1474 m was quiescent for nearly 75% of the 24 minutes viewing time, with the remainder mostly taken by gently skulling with the pectoral fins and four brief periods of burst swimming of up to 20 seconds each, usually when the ROV came close. Although only 36 seconds of whalefish behaviour are available, the 29/71 ratio of fin swimming to quiescence in this whalefish is surprisingly close to that of the anglerfish. Moore (2002) described three separate individuals of the anglerfish *Gigantactis* sp., filmed at different times on the same day, all drifting motionless, upside-down near the 5000 m bottom. They only swam when the ROV approached within 2-4 m, with a C-start and rapid swimming for 5-6 seconds, then slowed until they swam out of view in less than 15 seconds. One individual slowed its swimming rate after 5-6 seconds, even though the ROV moved closer to within 1 m. Moore (2002) suggested that a lack of stamina, consistent with the reduced musculature in all ceratioid anglerfishes, was responsible. The observed swimming behaviours of all three of these bathypelagic species correlate well with the low level of food and energy available at depths below 1000 m.

Additional DNA Results

In the whole mitogenomic tree of 15 specimens (figure 2*a*), sequence differences (% differences of the 15886 aligned sequences) ranged from 0.04-0.77% for "putative" conspecific species, 3.9-5.8% among closely related species (putative sister species) adjacent in the tree, and 14.6-26.2% among other species.

In the 16S rRNA tree of 36 specimens (figure S1), two larvae identified as *Parataeniophorus gulosus* and nine females of *Cestoma regani* cluster together, with one female more closely embedded between the two larvae. The species is known from all oceans (Paxton 1989); these larvae are from the north Atlantic (*P. gulosus* #1) and Pacific (*P. gulosus* #2), while eight females are from the north Atlantic (*C. regani* #1,3-9) and one from the Pacific (*C. regani* #2*).* The small genetic variation in this species does not appear to be correlated with distribution.

Female *Cetomimus* and *Gyrinomimus* are currently distinguished by two consistent morphological characters, the type of teeth in the jaws and the shape of the vomerine tooth patch (Paxton 1989). The genetic data indicate further work is required to elucidate the relationships of these two taxa.

DNA from tissue of the only alcohol-preserved specimen presumed to be the larva *Parataeniophorus brevis* was very difficult to sequence, with the DNA apparently degraded. We were unable to sequence the whole mitogenome, and therefore this taxon is excluded from figure 2a. In the 16S tree (figure S1) this larva is the sister taxon to *Cetostoma regani* and genetically distinct from this genus as well as *Danacetichthys, Gyrinomimus,*and *Cetomimus.*  With female *Procetichthys* linking with the larval *Mirapinna* and female *Ditropichthys* a probable link with larval *Parataeniophorus bertelseni,* and *Notiocetichthys* restricted to Antarctic waters, the only remaining female genera are *Cetichthys* and *Rhamphocetichthys*. The counts of vertebrae and dorsal- and anal-fin rays of *P. brevis* are virtually identical with *Rhamphocetichthys* and broadly overlap those of *Cetichthys*. Further specimens are needed to confirm a link.

*Additional Acknowledgements*

Numerous individuals have aided this project over the years, with specimens, tissues and/or data: K.T. Shao, H. Ho (ASIZ), S. Schiel (AWI, Bremerhaven), H. Endo (BSKU), W. Eschmeyer, D. Catania (CAS), N. Parin (IOANM), H. Senou (KPM), R. Lavenberg, J. Seigel (LACM), D. Bray (NMV), W. Richards (NOAA, Miami), K. Matsuura, G. Shinohara (NSMT), R. Rosenblatt, H. J. Walker (SIO), E. Fujii (Tokai Fisheries), A. Bucklin (UConn), J. Williams, S. Jewett (USNM), T. Pietsch, Z. Baldwin (UW), P. Wiebe (WHOI); images and curatorial assistance: M. McGrouther, A. Hay (AMS), S. Raredon, N. Schnell (USNM); research assistance: T. Trnski, D. Bray (AMS); translations: M. Motomura (AMS), W. Ivantsoff (Macquarie U.), A. Nonaka (USNM); and comments on the project and/ or manuscript: R. Britz, P. Konstantinidis (BMNH), B. Robison (MBARI), K. Hartel (MCZ), H. G. Moser (NOAA, La Jolla), R. McDiarmid (USGS), V. Springer, N. Schnell (USNM), R. Harbison (WHOI). R. Britz performed excellent partial dissections of cleared and stained specimens in 2003. J. Mounts offered instructional advice about Adobe Illustrator and E. Hilton kindly applied his imaging expertise to significantly improve the quality of the final figures. A Smithsonian Institution Short Term Visitor appointment to JP helped support his research at USNM.

Helfman, G. S., Collette, B. B. & Facey, D. E. 1997 *The diversity of fishes.* Malden, MA: Blackwell Science.

Inoue, J. G., Miya, M., Tsukamoto, K. & Nishida, M. 2000 Complete mitochondrial DNA sequence of the Japanese eel, *Anguilla japonica*. *Fish. Sci.* **67**, 118–125.

Inoue, J. G., Miya, M., Tsukamoto, K. & Nishida, M. 2001 A mitogenomic perspective on the basal teleostean phylogeny: resolving higher-level relationships with longer DNA sequences. *Mol. Phylogenet. Evol.* **20**, 275-85.

Ishiguro, N. B., Miya, M. & Nishida, M. 2003 Basal euteleostean relationships: a mitogenomic perspective on the phylogenetic reality of the "Protacanthopterygii". *Mol. Phylogenet. Evol.* **27**, 476-88.

Kawaguchi, A., Miya, M. & Nishida, M. 2001 Complete mitochondrial DNA sequence of *Aulopus japonicus* (Teleostei: Aulopiformes), a basal Eurypterygii: Longer DNA sequences and the higher-level relationships. *Ichthyol. Res.* **48**, 213–223.

Luck, G. D. & Pietsch, T. W. 2008 *In-situ* observations of a deep-sea ceratioid anglerfish of the genus *Oneirodes* (Lophiiformes: Oneirodidae). *Copeia* **2008**(2), 446-451. (DOI 10.1643/CE-07-075)

Maddison, W. P. & Maddison, D. R. 2000 *MacClade Version 3*. Sunderland: Sinauer Associates.

Marshall, N. B. 1960 Swimbladder structure of deep-sea fishes in relation to their systematics and biology. *Discovery Repts.* **31**, 1-128.

Marshall, N. B. 1971 *Explorations in the life of fishes.* Cambridge, MA: Harvard University Press.

Miya, M. & Nishida, M. 1999 Organization of the mitochondrial genome of a deep-sea fish, *Gonostoma gracile* (Teleostei: Stomiiformes): First example of transfer RNA gene rearrangements in bony fishes. *Mar. Biotechnol. (NY)* **1**, 416–426.

Miya, M. & Nishida, M. 2000 Use of mitogenomic information in teleostean molecular phylogenetics: a tree-based exploration under the maximum-parsimony optimality criterion. *Mol. Phylogenet. Evol.* **17**, 437-55.

Moore, J. A. 2002 Upside-down swimming behavior in a whipnose anglerfish (Teleostei: Ceratioidei: Gigantactinidae). *Copeia* **2002**(4), 1144-1146.

Paxton, J. R. 1989 Synopsis of the whalefishes (family Cetomimidae) with descriptions of four new genera. *Rec. Aust. Mus*. **41**(2), 135-206.

Stamatakis, A. 2006 RAxML-VI-HPC: maximum likelihood-based phylogenetic analyses with thousands of taxa and mixed models. *Bioinformatics* **22**, 2688-90.

Westneat, M. W., Hale, M. E., McHenry, M. J. & Long, J. H. 1998 Mechanics of the fast-start: muscle function and the role of intramuscular pressure in the escape behavior of *Amia calva* and *Polypterus palmas.* *J. Exp. Biol.* **201**, 3041-3055.

Yang, Z. 1994 Estimating the pattern of nucleotide substitution. *J. Mol. Evol.* **39**, 105-11.

Supplementary Figure S1. Maximum likelihood tree derived from analyses of partial 16S rRNA sequences from 36 specimens using RAxML ver. 7.0.4. Numerals beside internal branches indicate bootstrap values (only ≧50% shown) based on 1000 replicates. Scale indicates expected number of substitutions per site; red asterisks = larvae, blue asterisk = male.

Supplementary Video A. Female long-finned whalefish, *Cetostoma regani*. Segment 1, Monterey Canyon, CA, USA, 1450 m, 19 November 2004, MBARI T765, taken by ROV *Tiburon* from R/V *Western Flyer.* Camera, audio Karen Osborn, ROV Pilot, MBARI, 5 seconds; Segment 2, Monterey Canyon, CA, USA, 1640 m, 5 April 2006, MBARI T961, taken by ROV *Tiburon* from R/V *Western Flyer.* Camera, audio Bruce Robison, Senior Scientist, MBARI, 33 seconds; Segment 3, slow motion of last part of Segment 2, no audio, 25 seconds. Video courtesy of Monterey Bay Aquarium Research Institute; post production by David Rawlinson (AMS).
