# Supplementary figures and images for: Deep-sea mystery solved: astonishing larval transformations and extreme sexual dimorphism unite three fish families
Source: Biol Lett. 2009 Jan 20;5(2):235–9. doi: 10.1098/rsbl.2008.0722 (PMC2667197; doi:10.1098/rsbl.2008.0722)

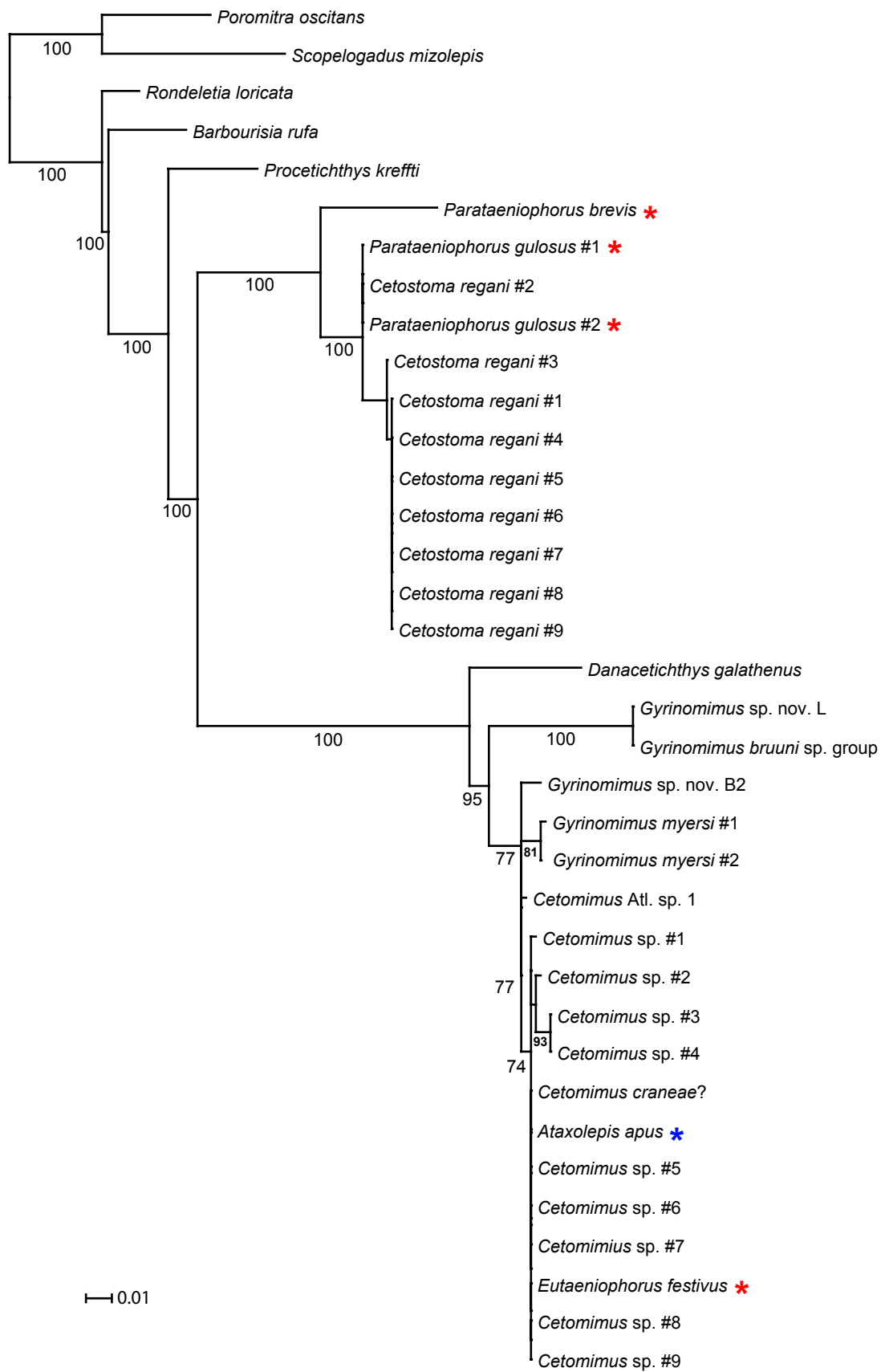

Supplement: Supplementary Figure S1. [file rsbl20080722s15.pdf]
